# Integrated Histological, Ultrastructural, Lectin and Immunohistochemical Characterization of the Senegalese sole (*Solea senegalensis*) Olfactory Rosettes: From Premetamorphic Larvae to Adult Individuals

**DOI:** 10.3390/ani16081144

**Published:** 2026-04-09

**Authors:** Dorinda Torres-Sabino, Albina Román, Paulino Martínez, Pablo Sanchez-Quinteiro

**Affiliations:** 1 Department of Anatomy, Animal Production and Clinical Veterinary Sciences, Faculty of Veterinary, Universidade de Santiago de Compostela, 27002 Lugo, Spain; dorinda.torres.sabino@usc.es; 2 Department of Zoology, Genetics and Physical Anthropology, Faculty of Veterinary, Universidade de Santiago de Compostela, 27002 Lugo, Spain; paulino.martinez@usc.es; 3 Electron Microscopy Unit, Research Infrastructures Area, Universidade de Santiago de Compostela, 27002 Lugo, Spain; a.roman@usc.es

**Keywords:** olfactory epithelium, flatfish, olfactory sensory neurons, immunohistochemistry, transmission electron microscopy

## Abstract

The olfactory system plays a crucial role in mediating fish behaviour, including reproduction. Senegalese sole (*Solea senegalensis*) is an important aquaculture flatfish species in Europe, in which reproductive dysfunction in captive males has been linked to potential alterations in chemical communication. Despite the expanded repertoire of olfactory receptor genes described for this species, detailed information on the cellular organization of its olfactory organs remains limited. This study provides a comprehensive histological, immunohistochemical, lectin-histochemical, and ultrastructural characterization of the olfactory rosettes of *S. senegalensis* across multiple life stages, including premetamorphic larvae, fry, juveniles and adults. Although the olfactory organs undergo substantial structural changes following metamorphosis, differentiated and functionally active olfactory sensory neurons (OSNs) are already present in premetamorphic larvae. Subsequently, two epithelial regions were distinguished along the olfactory lamellae: a sensory epithelium containing abundant OSNs and supporting cells, and a nonsensory epithelium dominated by goblet and other secretory cells. Ciliated and microvillous OSNs were distinguished from 60 dph onward based on morphological and ultrastructural features and supported by immunoreactivity with CR, CB, Gγ8 and PGP. Crypt cells showed immunolabelling with S100, NSE and CYK8. Furthermore, lectin histochemistry revealed ontogenetic changes in epithelial glycoconjugates, with early diffuse binding patterns evolving into stratified and region-specific distributions. Overall, these results demonstrate the structural and functional complexity of the olfactory epithelium in *S. senegalensis*, significantly enriching the limited available morphological and neurochemical information on the species.

## Simple Summary

The sense of smell plays an essential role in fish behaviour, including finding food, recognizing other individuals, and reproduction. Senegalese sole is an important fish species for aquaculture in Europe, but males raised in captivity show reproductive problems that may be related to changes in chemical communication through olfaction. However, little is known about how the olfactory organ of this species is organized at the cellular level. We found that the olfactory organ undergoes important structural changes during development, but functional sensory cells are already present at very early life stages. Notably, a well-developed and highly complex organ was observed, including several types of sensory cells responsible for odour detection. Our findings provide new information about how the olfactory organ of this species is organized and develops, which may help improve our understanding of chemical communication and contribute to better management of reproduction in aquaculture.

## 1. Introduction

The olfactory system is essential for fundamental behaviours across all vertebrate taxa [1,2]. In tetrapods, this chemosensory capacity is largely mediated by two interconnected subsystems: the main olfactory system and the vomeronasal system [3,4,5]. In contrast, except for lungfishes [6,7], the fish olfactory system relies on a unique sensory organ composed of a paired, multilamellar structure commonly known as olfactory rosettes. The olfactory rosettes are located rostromedial to the eyes, within an olfactory chamber in direct contact with the external environment through the nostrils [8].

The olfactory epithelium (OE) is arranged on both sides of the multiple lamellae that emerge from a central raphe, hosting a diverse array of sensory and nonsensory cell types [9,10]. A large proportion of fish OE consists of olfactory sensory neurons (OSNs), mainly including ciliated (cOSNs), microvillous (mOSNs) and crypt cells (CCs), along with supporting cells, basal cells and mucus-secreting goblet cells that collectively maintain epithelial structure and function [10]. Each OSN appears to express a single olfactory receptor responsible for the detection of a specific range of odorants [2,11]. Accordingly, fish olfactory capabilities encompass the detection of a wide range of waterborne chemical cues that mediate a broad spectrum of fundamental behaviours, such as food detection, reproduction, prey localization and migration [12,13,14]. Over the past decades, anatomical and functional studies in fish have revealed a highly developed and structurally complex sensory system, supporting their central role in chemical sensing across aquatic environments [15,16,17,18].

Broad morphological and functional diversity exists in the olfactory rosettes of teleost fish and elasmobranchs, particularly in the number, size, and arrangement of lamellae, reflecting species-specific ecological adaptations and influencing olfactory sensitivity and efficiency [19,20]. In demersal species, such as flatfish (Order Pleuronectiformes), olfaction has been proposed as a sensorial alternative to vision for the reduced visual cues in the seabed environment, characterized by low light irradiance and high sediment concentration [21,22]. Flatfish possess a unique olfactory anatomy, with an upper olfactory rosette on the ocular side exposed to the open water column, and a lower olfactory rosette on the blind side in contact with interstitial water [8]. This asymmetrical organization arises after a dramatic metamorphosis process, which transforms pelagic larvae into bilaterally asymmetric specimens with a demersal lifestyle [23,24]. In turbot, olfactory organs lack lamellae at premetamorphic stages, although the OE appears to be functionally active at this early life stage [25,26].

Senegalese sole (*Solea senegalensis*) is a promising flatfish species for European aquaculture [27]. However, the establishment of an efficient captive breeding system remains one of the major constraints limiting the expansion of the industry, associated with altered spawning and courtship behaviours observed in captive-bred males [28,29]. In fish, olfactory cues play a key role in mediating reproductive behaviours, and it has been demonstrated that chemical signals released by ovulating females can directly induce male courtship responses [30,31]. Given the central role of olfactory chemoreception in fish reproduction, several studies aimed at understanding the reproductive dysfunction in this species have been conducted, including transcriptomic approaches characterizing gene expression profiles in the olfactory organs [32,33,34]. In this context, *S. senegalensis* has been shown to possess a highly specialized olfactory system, with an expanded olfactory receptor gene repertoire compared to other flatfish species [35,36]. However, morphological information on the olfactory system of *S. senegalensis* remains limited. To date, only brief histological descriptions have been reported, focusing on premetamorphic larvae [37] and adult individuals [32,34]. Therefore, a comprehensive morphological and neurofunctional characterization of the OE is still needed to elucidate the mechanisms underlying olfactory chemoreception in *S. senegalensis*.

The present study aims to provide a detailed morphological and neurochemical characterization of the OE throughout life stages, from premetamorphic larvae to adult *S. senegalensis* individuals. To this end, a comprehensive anatomical description of the olfactory rosettes, combining histological, lectin-histochemical and immunohistochemical approaches, was addressed. In addition, an ultrastructural analysis of the adult OE was conducted to refine the description of the epithelial organization. Overall, our study reveals a structurally complex and dense cellular epithelium even at larval stages, composed of a heterogeneous population of OSNs, supporting nonsensory cells, goblet cells and basal cells. Finally, we provide a comprehensive histochemical characterization based on a wide panel of lectins and antibodies commonly used in olfactory studies that allow a detailed visualization of cellular components, distribution patterns, and structural organization within the olfactory epithelium. The well-developed and specialized olfactory organ, since premetamorphic stages, supports the key role of olfaction in *S. senegalensis* behaviour, offering valuable insights to understand flatfish chemoperception.

## 2. Materials and Methods

### 2.1. Animal Sampling

Individuals of different life stages were used in this study, including 12 premetamorphic larvae of 5 days post-hatching (dph), six 60 dph fry, six 96 dph fry, six 126 dph fry, three 10-month-old (10 mo) juveniles and three 27-month-old (27 mo) adults were provided by Stolt Sea Farm (Cervo, Spain), where they were reared at standard conditions [38]. These broad life stage samples were selected to comprehensively examine the development of the olfactory organs in *S. senegalensis*. Fish were anesthetized to unconsciousness by immersion in ice (hypothermia) and subsequently euthanized by decapitation in accordance with EU guidelines (Directive 2010/63/EU). Afterwards, the olfactory chambers, where the olfactory organs are located, were accessed through an incision between the two nostrils after removing the skin. To facilitate dissection, the surrounding connective tissue within the olfactory chamber was removed and then the olfactory nerves were cut for the extraction of the whole olfactory organ. In the case of 126 dph individuals, the dissection of the olfactory organs was performed under a surgical microscope, Zeiss OPMI 1 (Oberkochen, Germany). Given the small size of the 60 and 96 dph fry, the whole head was fixed. Finally, the whole individual was fixed in the case of premetamorphic larvae due to the small size and fragility of the olfactory organ.

Samples were immediately fixed by immersion in Bouin’s fixative solution for 24 h. Afterwards, they were transferred to 70% ethanol. In all cases, the histological procedures were performed using paraffin embedding and no decalcification was needed. No decalcification was performed in any of the individuals, as sectioning could be carried out successfully without this procedure, thereby minimizing tissue manipulation and preserving the histological and neurochemical properties of the olfactory tissue. Each sample was cut using a rotatory microtome (Leica Reichert Jung, Wetzlar, Germany) with a thickness between 4 and 7 μm. For routine histological examination, sections from all samples were stained with hematoxylin and eosin (H&E) to differentiate tissue components. Briefly, sections were first stained with hematoxylin for 2 min to label nuclear components, followed by thorough washing. Cytoplasmic and extracellular components were then stained with eosin for 5 min. After staining, sections were washed, dehydrated through a graded ethanol series, and mounted for microscopic examination.

### 2.2. Ultrastructural Study

Olfactory rosettes from two adult individuals were dissected and cut into approximately 1 mm^3^ tissue blocks prior to immersion in a fixative solution consisting of 2.5% buffered glutaraldehyde in 0.1 M cacodylate buffer (pH 7.3) for 4 h at 4 °C. Post-fixation was performed in 1% osmium tetroxide prepared in the same buffer. Following standard procedures, tissues were dehydrated through a graded ethanol series and embedded in epoxy resin (Embed 812, Electron Microscopy Sciences, Hatfield, PA, USA) [39]. Thick sections (0.5 um) were cut from the epoxy blocks, mounted on glass slides, stained with Toluidine blue and imaged using an AxioCam MRc 5 (Zeiss, Oberkochen, Germany). Ultrathin sections were counterstained with uranyl acetate and lead citrate, and then examined using a JEOL JEM-1011 transmission electron microscope (TEM, Akishima, Japan).

### 2.3. Immunohistochemistry (IHC) Techniques

Immunohistochemistry analysis (IHC) was conducted on olfactory rosette sections from all stages analyzed, following the same strategy as in [26]. Briefly, a suppression of endogenous peroxidase activity was performed to avoid nonspecific staining using a solution of 3% H_2_O_2_ in distilled water. Afterwards, residues were eliminated from the slides by washing in 0.1 M phosphate buffer (PB, pH 7.2). 2% bovine serum albumin (BSA) was used for 30 min to block nonspecific bindings. Then, the primary antibody was added to each section to be incubated overnight at 4 °C. Three washes using PB were performed to eliminate the excess of antibody. Depending on the specific blocking agent employed, the slides were then incubated for 30 min with the CRF Anti-Polyvalent HRP Polymer (ScyTek, Logan, UT, USA). Next, a 10 min rinse in 0.2 M Tris–HCl buffer at a pH of 7.61 was performed. A diaminobenzidine (DAB) chromogen was used for visualization, using a solution of 0.003% H_2_O_2_ and 0.05% DAB in 0.2 M Tris-HCl buffer, generating a brown deposit in the slide when positive binding. Positive controls were performed with the same procedure in species with well-known immunoreactions, whereas negative controls included the omission of the primary antibody. The employed antibodies are summarized in Table 1.

### 2.4. Histochemical Labelling with Lectins (Lectin-HC)

Lectins are specialized proteins not derived from immunological processes that contain carbohydrate-recognition domains that bind non-covalently to specific terminal sugars on tissues, leading to the formation of glycoconjugates [40]. Lectins are widely used for studying the olfactory organs of both fish [26,41,42,43] and mammals [44]. We followed the same procedure as in [26]. Briefly, non-specific bindings were blocked with 2% BSA, then biotinylated lectins were added to be incubated overnight. Sections were subsequently incubated for 90 min in an avidin-biotin-peroxidase (ABC) solution (Vector Laboratories, Burlingame, CA, USA). The complex binds to the lectin during incubation, enhancing the peroxidase reaction. Signal development was performed as described for IHC. The employed lectins are summarized in Table 2.

### 2.5. Acquisition of Images and Digital Treatment

Digital images were captured using an Olympus SC180 digital camera connected to an Olympus microscope model BX50 (Hachioji, Japan). To achieve maximum definition, some of the presented photomicrographs are the outcome of combining a mosaic of various images, using an automatic stitching software (PTGui v12.27, Rotterdam, The Netherlands). Adobe Photoshop CS4 (Adobe Systems, San Jose, CA, USA) was used. Image processing was limited to uniform brightness and contrast adjustments corresponding to white balance correction, applied to the entire image to normalize background illumination across samples. No selective enhancements or alterations affecting signal interpretation were performed. Photostitching was used to combine multiple images from the same preparation into a single high-resolution composite image, without altering the original signal.

## 3. Results

We present the results of the histological characterization of the *S. senegalensis* olfactory organs across various life stages, including premetamorphic larvae (5 dph), fry (60 dph, 96 dph, 126 dph), juvenile (10-month-old (10 mo)) and adult (27-month-old (27 mo)) individuals. A detailed description of the olfactory organs is provided following the normal developmental order, from premetamorphic to adult individuals.

### 3.1. Microscopic Study of the S. senegalensis Olfactory Organs Across Different Life Stages

**Figure 1:**
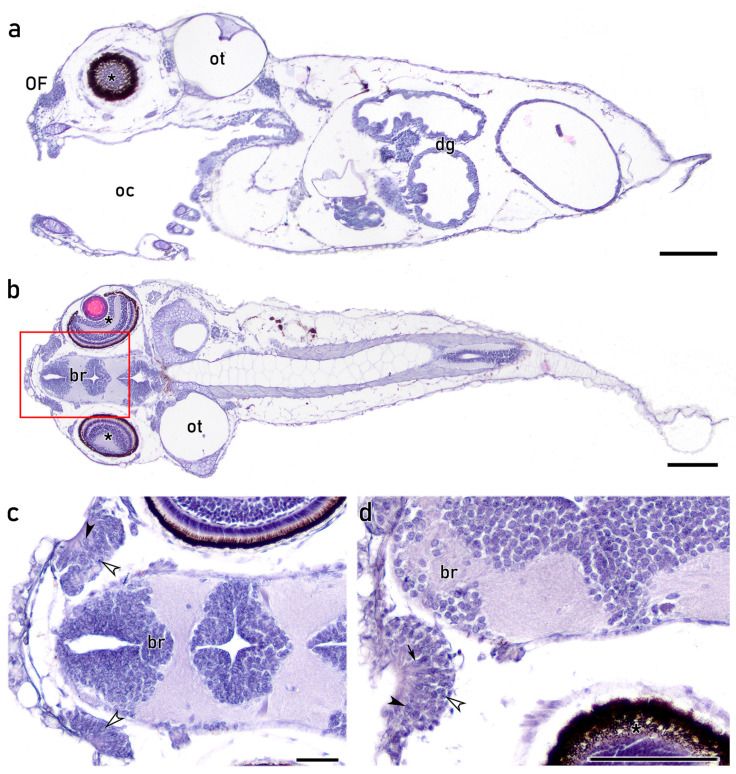
Histology of premetamorphic larvae (5 dph) stained with Hematoxylin-Eosin (H&E). (**a**) Sagittal section of the entire larva showing structures such as the oral cavity (oc), digestive tract (dg), eye (asterisk) and otic capsule (ot). The olfactory fossae (OF) appear as a “c” shape epithelium located rostrally to the eyes and in direct contact with the environment. (**b**) Parasagittal section of the entire larva. The two OFs are visible, located at both sides of the head, close to the brain (br) and the eyes (asterisks). Ots are visible caudally to the eyes. At this level, both OFs possess direct communication with the environment. (**c**) Inset from (**b**) showing both OFs located close to the br. Despite lacking lamellae, the olfactory epithelium (OE) comprises a diverse cell population, including scattered nuclei located in a more apical region (black arrowhead) and rounded nuclei in basal positions (open arrowheads). (**d**) The OE comprises diverse cell morphologies, including spindle-shaped cells along the epithelium (black arrow), rounded apical nuclei (black arrowhead) and rounded basal cells (open arrowhead). oc, oral cavity; dg, digestive tract. Scale bars: 100 μm (**a**,**b**); 50 μm (**c**,**d**).

The premetamorphic histological study offers a complete image of the individual, where the olfactory organs differ greatly in their morphology when compared to later stages (Figure 1a,b). At this stage (5 dph), the organs are known as olfactory fossae (OFs), consisting of a circular pit of epithelium lacking the typical multilamellar shape (Figure 1a–d). The OFs display a relatively large size when compared to the head, where they occupy an anterodorsal position, close to the eyes (Figure 1b). The OFs are positioned close to the forebrain, where the olfactory nerves project (Figure 1b–d). In parasagittal sections stained with H&E, the OFs exhibit a characteristic “c” shape, showing direct communication to the external environment, with no skin covering the OFs (Figure 1b,c). Despite the structural differences between OF and the multilamellar rosette, the OFs encompass a conglomerate of cells showing a remarkable diversity in cell morphology, including oval and rounded nuclei, with some cells displaying spindle-shaped processes that reach the apical regions of the OE (Figure 1c,d). Although nuclei seem to be predominantly located in central and basal areas, scattered apical nuclei are observed (Figure 1c). Notably, no secretory cells were observed on the premetamorphic OE.

**Figure 2:**
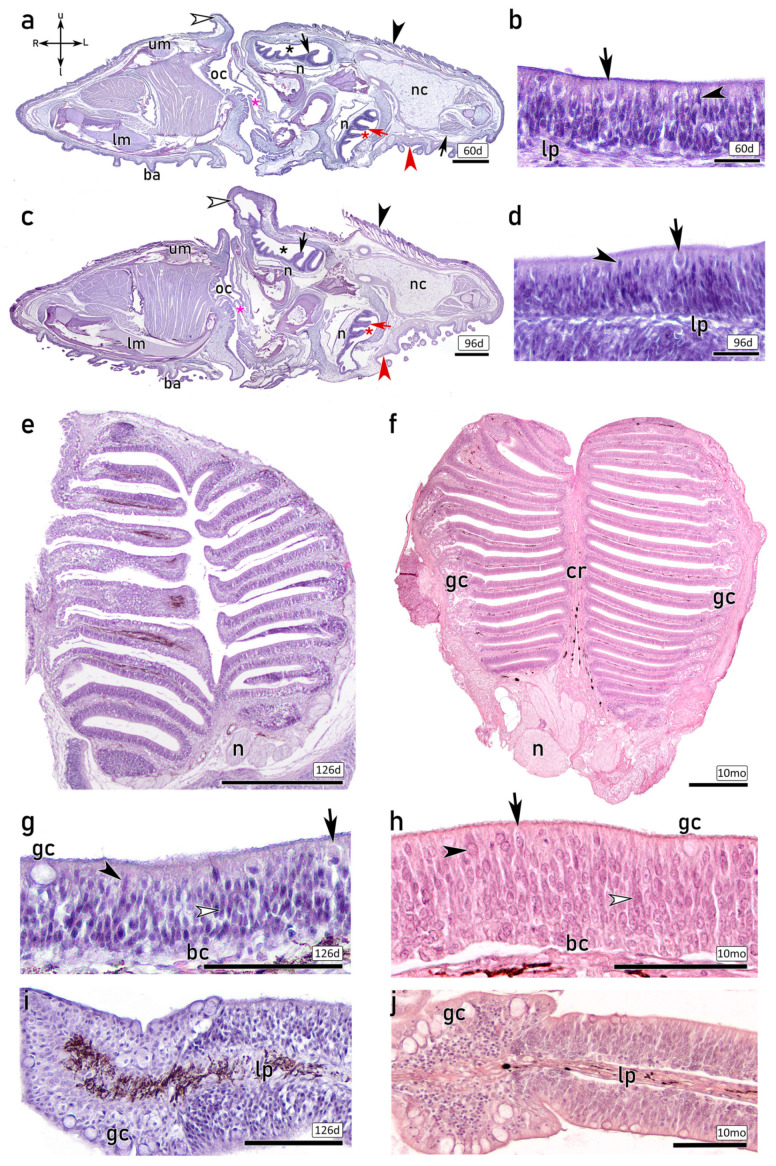
Histological study of the olfactory rosettes of 60 dph (**a**,**b**), 96 dph (**c**,**d**), 126 dph (**e**,**g**,**i**) and 10-month-old (10 mo) (**f**,**h**,**j**) individuals stained with Hematoxylin-Eosin (H&E). (**a**,**c**) Transversal section of the complete head showing the anatomy of both upper and lower rosettes within their olfactory chambers (asterisks) on a 60 dph (**a**) and on a 96 dph (**c**). In both sections (**a**,**c**), the upper olfactory chambers (black asterisks) hosting the upper rosettes (black arrows) and the lower olfactory chambers (red asterisks) and lower rosettes (red arrows) displaying lamellae and nerve bundles (n) are visible. The upper skin (black arrowhead) shows a skin flap (open arrowhead), and the lower skin (red arrowhead) displays barbels (ba). Other structures are indicated, such as upper mandible (um) and lower mandible (lm), together with oral cavity (oc), dorsal palatal recess (pink asterisk) and neurocranium (nc). (**b**,**d**) Sections of the olfactory epithelium (OE) of 60 dph (**b**) and a 96 dph (**d**) fry, showing a highly cell-dense OE, with rounded and oval nuclei mainly distributed in basal and central regions. Some strong basophilic oval nuclei are located close to the luminal surface (black arrowheads). The luminal surface is covered with cilia projecting from both sensory and nonsensory cells. Scattered CCs, surrounded by slender supporting cells, show globose cell bodies and nuclei located in the basal part of the cell body (arrows in (**b**,**d**)). The basal lamina delimits the OE and the lamina propria (lp). (**e**) Horizontal section of the complete upper olfactory rosette in a 126 dph fry. Lamellae are parallelly positioned. The OE arises on both sides of the laminal propria of each lamella. Nerve bundles (n) are visible, and also black melanin deposits. (**f**) Complete horizontal section of the upper olfactory rosette of a 10 mo juvenile, showing lamellae emerging from both sides and parallelly from the central raphe (cr). The OE arises from both sides of each lamella. The axons projecting from the olfactory neurons converge in the lamina propria to form large nerve bundles (n). Melanin deposits are visible. The peripheral region of each lamella shows a distinct epithelial pattern, which mainly consists of goblet cells (gc). (**g**) 126 dph OE shows numerous highly basophilic nuclei concentrated in the basal and central regions of the epithelium. In the most apical region, in direct contact with the luminal surface, large and rounded gc are present. Abundant cilia cover the luminal surface. Some oval basophilic nuclei are located in the most apical region (black arrowhead). Isolated sensory crypt cells are observed (arrow), characterized by a globose cell body with the nucleus located in the basal part of the soma and slender projections toward the basal region of the OE. Spindle-shaped sensory cells with oval nuclei are visible in central regions, displaying narrow dendrites projecting cilia to the lumen (open arrowhead); and rounded basal cells (bc) are found in the most basal region of the OE. (**h**) The OE of a 10 mo juvenile displays a pseudostratified columnar and densely cellular organization. Apically, large and rounded gc are observed, together with globose somata resembling a CC (arrow) and other rounded cells whose processes reach the luminal surface (black arrowhead). In central regions, rounded and oval nuclei are located (open arrowhead). They belong to spindle cells with long and densely packed processes extending to the apical region. Basally, small, rounded bc are visible. (**i**,**j**) Peripheral region of the lamellae of 126 dph (**i**) and 10 mo (**j**) individuals, where the characteristic stratified nonsensory epithelium is found. Numerous large and rounded gc are visible in the apical regions, while deeper layers show abundant non-neural rounded cells. Melanin deposits are visible in the lp. u, upper; l, lower; R, right; L, left; bc, basal cells; lp, lamina propria; cr, central raphe; n, nerve bundles; gc, goblet cells; CC, crypt cell; 10 mo, 10 month-old. Scale bars: 500 μm (**a**,**c**,**e**,**f**); 25 (**b**,**d**); 50 μm (**g**–**j**).

The microscopic study of the olfactory organs continued with the examination of 60 dph (Figure 2a,b) and 96 dph (Figure 2c,d) fry, including transversal sections of their complete heads stained with H&E. The histological study reveals extensive remodelling of the olfactory organs during metamorphosis, after which they acquire the anatomical organization that persists throughout later life stages.

In both stages (60 and 96 dph), the upper rosette occupies the ocular side of the olfactory chamber, where both eyes are situated, although they are not visible at this histological level. The lower rosette is located within the blind side olfactory chamber. Both rosettes exhibit a typical multilamellar shape, with no disparities between stages beyond slight size differences (Figure 2a,c). Nerve bundles are visible emerging from the basal region of the olfactory rosettes, where they converge to form the olfactory nerves. At these stages, the OE already consists of a complex and highly cell-dense pseudostratified columnar epithelium, including rounded and oval nuclei predominantly positioned in the central and basal regions. The OE encompasses olfactory sensory neurons (OSNs) and nonsensory supporting cells of varying morphologies, together with apical large goblet cells responsible for mucus secretion and basal rounded cells. The luminal surface is covered by abundant cilia projecting from both sensory and nonsensory cells. Different OSN morphologies are distinguishable, including cells resembling sensory crypt cells (CCs), characterized by an apical position, globose soma and basally located nucleus. Furthermore, spindle-shaped cells projecting their dendrites to the lumen are observed (Figure 2b,d).

The larger size of the 126 dph individuals allows for a complete dissection of the olfactory rosettes under a surgical microscope. Consequently, horizontal sections of the whole olfactory rosette stained with H&E are presented on both 126 dph fry and 10 mo juvenile individuals. As development progresses, the olfactory rosette increases in size. Histological sections reveal the overall organization of the organ, characterized by a multilamellar structure with lamellae arranged in parallel (Figure 2e,f). Given the superficial level of the section shown in the 126 dph individual (Figure 2e), the central raphe is not observed. In contrast, a thick central raphe of connective tissue from which the lamellae emerge, dividing the two symmetric parts of the olfactory rosette, is observed in the 10 mo individual (Figure 2f). Melanin deposits are present in the lamina propria. Consistent with earlier developmental stages, the axons from the OSNs merge into prominent nerve bundles (Figure 2e,f).

H&E staining of the OE reveals its epithelial organization, allowing for the discrimination of distinct cell morphologies and their spatial arrangement across the OE. Although cells are densely intermingled within the pseudostratified OE, distinct cell morphologies are distinguishable, displaying a similar pattern in both the 126 dph and the 10 mo individuals (Figure 2g–j). The apical region of the OE displays lower nuclear density when compared with deeper layers. Centrally located rounded cell bodies projecting their dendrites to the luminal surface resemble microvillous sensory neurons (mOSNs), whereas globose CC in apical positions, and ciliated sensory neurons (cOSNs) in deeper layers are characterized by slender dendrites extending to the lumen (Figure 2g,h). Close to the basal lamina, which delimits the OE and the lamina propria, rounded basal cells are located. The luminal surface, covered by cilia, shows large mucus-secreting goblet cells, which are present at higher density in the nonsensory epithelium located at the peripheral region of the lamellae (Figure 2i,j). This peripheral nonsensory area consists of a stratified epithelium, including small and rounded cells with basophilic nuclei at deeper layers, together with abundant apical goblet cells.

**Figure 3:**
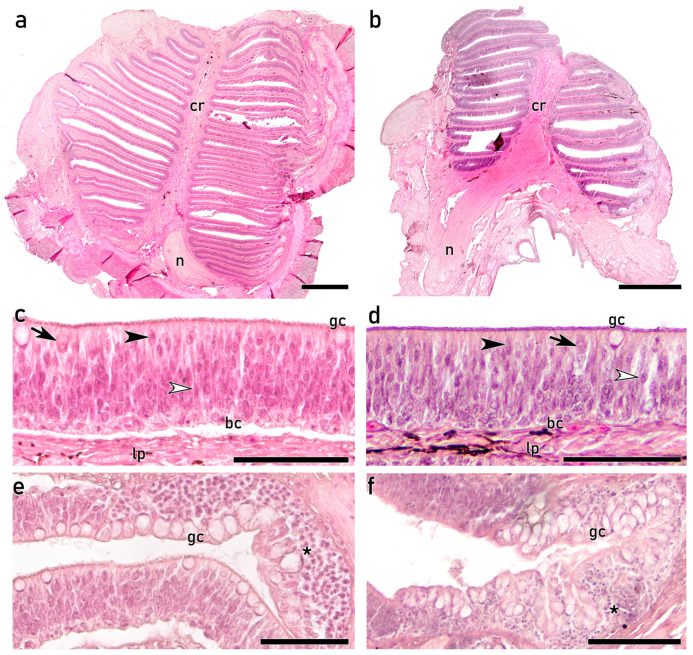
Histological study of the upper (**a**,**c**,**e**) and lower (**b**,**d**,**f**) rosettes in adult *S. senegalensis* stained with Hematoxylin-Eosin (H&E) (**a**,**b**). Horizontal central section of the complete upper (**a**) and lower (**b**) rosettes. The bigger upper rosette shows a greater number of lamellae, which emerge in parallel in both rosettes from a prominent central raphe (cr). Each lamella displays olfactory epithelium (OE) arising from both sides of the lamina propria. Axons emerging from the sensory neurons converge in the lamina propria, forming large nerve bundles (n). (**c**,**d**) Highly dense pseudostratified OE, displaying intermingled cells with distinguishable morphologies and spatial arrangements. Apically, globose crypt cells (CCs) with broad cytoplasm (arrows) and cells with oval nuclei projecting narrow processes are observed (black arrowheads). In addition, mucus-secreting goblet cells (gb) are scattered through the OE. In central areas, spindle-shaped cells with long and densely packed processes that reach the apical surface are visible (open arrowheads). The luminal surface is covered by cilia. In the most basal region of the OE, rounded basal cells (bc) are shown. The basal lamina delimits the OE from the lamina propria (lp), where melanin deposits are observed. (**e**,**f**) The peripheral region of each lamella displays a distinct epithelium, characterized by numerous prominent gb in apical regions and rounded small cells in deeper layers (asterisks). cr, central raphe; n, nerve bundles; lp, lamina propria; gc, goblet cells; CC, crypt cell. Scale bars: 500 μm (**a**,**b**); 50 μm (**c**–**f**).

For the examination of the olfactory rosettes on adult individuals, both the upper and lower olfactory rosettes were dissected, and thus, a morphological description of each rosette is provided. Complete horizontal sections through the central region of the olfactory organs reveal the olfactory rosettes at their maximum development, showing multiple lamellae emerging in parallel from a thick central raphe, and covered by OE on both sides (Figure 3a,b). The upper olfactory rosette is bigger and displays a greater number of lamellae than the lower rosette. Beyond these differences, both rosettes exhibit a similar epithelial organization, consisting of a pseudostratified epithelium formed by densely packed cells with diverse morphologies and spatial arrangements along the epithelial surface (Figure 3c–f). The apical surface of the OE is covered by cilia projecting from both sensory and nonsensory cells. Distinct morphologies of OSNs are observed along the OE. Within the most apical part of the OE, cells ranging from oval to rounded bodies featuring large processes that extend to the lumen are observed, together with globose cell bodies typical of CCs. In deeper layers, spindle-shaped cells displaying slender processes typical of cOSNs that reach the lumen are visible (Figure 3c,d). These OSNs are often difficult to discriminate from the supporting intermingled nonsensory cells. Basal rounded cells are located close to a well-defined basal lamina, which delimits the OE from the lamina propria. Furthermore, secretory goblet cells in apical positions are distributed along the entire lamellae, although mostly concentrated in the outer nonsensory areas, together with abundant small and rounded cells in deeper layers (Figure 3e,f).

### 3.2. Ultrastructural Study of the Adult Olfactory Epithelium (OE)

**Figure 4:**
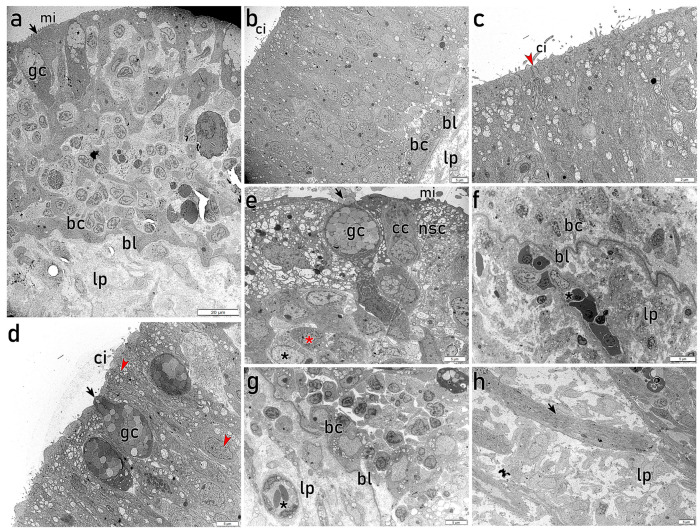
Transmission electron microscopy of diverse zones of adult olfactory epithelium (OE). (**a**) Image of the outermost part of a lamella, extending from the basal lamina (bl) to the apical surface of the OE. The apical region is predominantly occupied by electron-dense cells (arrow) with wide cell bodies in direct contact with the lumen, from which they project multiple microvilli (mi). Secretory goblet cells (gc) are also visible. Deeper layers show polyhedral cells arranged in clusters of predominantly electron-lucent cells, surrounded by rings of irregularly shaped electron-dense cells. Basally, rounded and small basal cells (bc) are visible. The lamina propria consists of loose connective tissue. (**b**) Photomicrograph of the OE from the bl to the luminal surface, showing cilia (ci) projecting from both sensory and nonsensory cells. Oval and rounded nuclei are predominantly positioned in central and basal areas, where bc are located. Bl clearly delimits the OE and lp. (**c**) Higher magnification view of the epithelial surface showing ci projecting from both sensory and nonsensory ciliated cells. A slender dendrite projecting ci to the lumen is visible (red arrowhead). Abundant electron-lucent secretory granules occupy the apical part of supporting cells. (**d**) Apical region of a central lamellar area, showing abundant gc directly secreting into the lumen (arrow). A cell displaying a rounded nucleus located basally, with a dendrite projecting toward the lumen is observed (red arrowheads). (**e**) Photomicrograph of the OE showing apical nonsensory cells (nsc) bearing mi and containing electron-lucent secretory granules. A globose cell body with a basal nucleus is consistent with the morphology of a crypt cell (CC). A large gc with an electron-dense apical projection directly releasing its content into the lumen (arrow) is visible. In deeper layers, small cells with oval to irregularly shaped nuclei show either high electron-density (red asterisk) or high electron-lucent (black asterisk). (**f**) Detail of the basal part of the OE, where electron-dense bc are separated from lp by a thick bl. Within the lp, irregularly shaped, highly electron-dense cells are observed (black asterisk), consistent with macrophage-like cells. (**g**) Bc is closely located to the thick bl, which separates the OE from lp, containing connective tissue elements, blood vessels (asterisk) and scattered cells. (**h**) Micrograph of the lp showing a large axon (arrow) projecting from olfactory sensory cells, together with collagen fibres and associated connective tissue components. mi, microvilli; gc, goblet cells; bc, basal cells; bl, basal lamina; lp, lamina propria; ci, cilia; CC, crypt cell; nsc, nonsensory cell.

**Figure 5:**
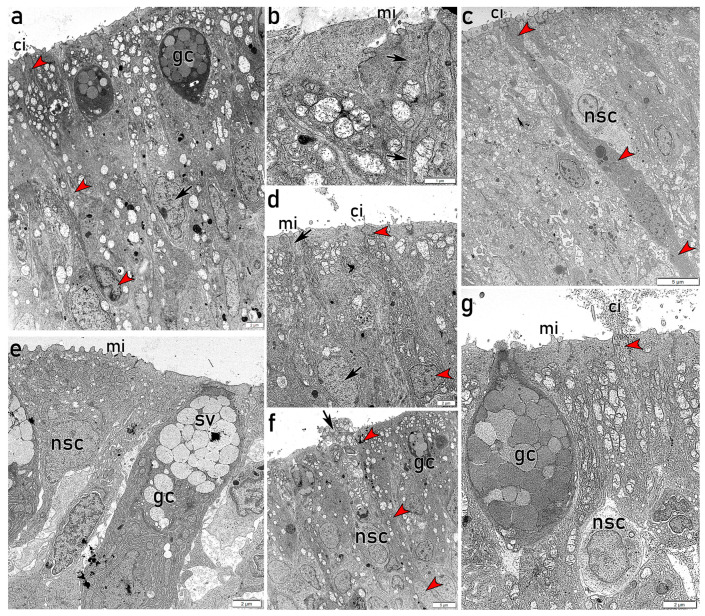
Transmission electron microscopy micrographs illustrating the diversity of cell types observed across the olfactory epithelium (OE) in adult individuals. (**a**) Photomicrograph showing an electron-dense ciliated olfactory sensory neuron (cOSN) (red arrowheads) with an elongated and irregular cell body projecting a slender dendrite toward the lumen, where cilia (ci) are visible. The periphery of the nucleus shows abundant granular heterochromatin. Apically, goblet cells (gc) filled with secretory vesicles are located. Cell bodies and nuclei of diverse morphologies are visible, including cells located in central areas of the OE with a rounded nucleus positioned basally within a pear-shaped cell body, typical of microvillous OSN (mOSN) (black arrow). (**b**) Detail micrograph of a slender pear-shaped dendrite that narrows abruptly to form the axon (black arrows). (**c**) An electron-dense elongated cOSN projecting a narrow dendrite and ci into the lumen (red arrowheads), surrounded by electron-lucent nonsensory cells (nsc) with nuclei located more apically. (**d**) Detail micrograph illustrating the typical slender dendrites of different types of OSN projecting ci and mi, including both mOSNs (black arrows) and cOSNs (red arrowheads). In the latter, abundant heterochromatin is evident within the nucleus. (**e**) In the outermost part of the lamella, gc containing abundant secretory vesicles (sv) are observed. In addition, apical electron-dense nsc display wide luminal projections with numerous short mi. (**f**) High cellular density is evident, including rounded nuclei and more electron-lucent nsc. In a more basal position, oval nuclei of electron-dense cells projecting their dendrites are visible (red arrowheads). In apical positions, a cell actively secreting electron-lucent secretory granules into the lumen (arrow) is observed. (**g**) A large globose gc is apically positioned, directly secreting into the lumen. An electron-lucent nsc with a rounded nucleus surrounds a narrow dendrite bearing abundant ci (red arrowhead). ci, cilia; gc, goblet cells; mi, microvilli; nsc, nonsensory cell; sv, secretory vesicles.

Given the small cell size, high cellular diversity, faint staining of cellular processes and scarce available information in flatfish, the OE characterization based solely on routine histological study with H&E is limited. Thus, to enhance the OE description, we present here the results of the transmission electron microscopy (TEM) of ultrathin sections of the OE on adult individuals (Figure 4 and Figure 5). While routine histology provides an overview of the different cell types, TEM enables a more detailed epithelial characterization, allowing the discrimination among distinct subpopulations of both OSNs and nonsensory cells.

The different epithelial areas analyzed by TEM display distinctive features and cell distributions, consistent with the images of the histological study. The peripheral part of each lamella exhibits a nonsensory epithelium with abundant apical goblet cells filled with secretory vesicles, together with electron-dense nonsensory microvillous cells. Deeper layers show a high density of polyhedral cells arranged in clusters of predominantly electron-lucent cells, surrounded by rings of large, irregularly shaped electron-dense cells (Figure 4a). In contrast, the central and inner parts of the lamellae display a pseudostratified OE with nuclei predominantly located in central and basal positions within cells of varying electron-density. Some of these cells show spindle-shaped projections that reach the lumen, where cilia are observed (Figure 4b). The apical region of the OE shows abundant secretory electron-lucent granules within nonsensory cells and slender dendritic processes projecting from cOSNs (Figure 4c,d), together with apical goblet cells (Figure 4d,e). Globose cell bodies in apical position and surrounded by nonsensory cells are consistent with the morphology of CCs (Figure 4e). The central and basal layers of the OE exhibit cells with diverse morphologies and electron-densities (Figure 4e–g), including rounded basal cells close to a thick basal lamina (Figure 4f,g). TEM allows a clear characterization of the lamina propria, revealing large axons projecting from OSNs (Figure 4h), abundant collagen fibres, fibroblasts and blood vessels. Furthermore, highly electron-dense polyhedral cells are observed in the lamina propria. Based on their ultrastructural features and irregular morphology, these cells are consistent with macrophage-like cells, although their precise identity cannot be conclusively determined.

TEM allows the examination of individual cell subtypes, enabling the study of specific cellular paths across the OE. Spindle-shaped electron-dense cells projecting long dendrites bearing cilia toward the lumen are characteristic of cOSNs, with nuclei displaying granular heterochromatin in the periphery (Figure 5a,c). These cOSNs can extend across the entire thickness of the epithelium (Figure 5c). Notably, apical dendrites occasionally give rise to narrow axon-like processes, as shown in Figure 5b. Other cells show slightly lower electron-density and oval bodies that also project narrow dendrites to the lumen, although bearing short microvilli, characteristic of mOSNs (Figure 5d). Generally, the supporting non-sensory cells surrounding OSNs appear more electron-lucent, showing variable morphologies and projecting both cilia and microvilli (Figure 5c,f,g). In the peripheral region of the lamellae, electron-dense nonsensory cells with abundant microvilli are located apically (Figure 4a and Figure 5e). Interestingly, abundant electron-lucent secretory granules are observed in the apical part of these nonsensory cells, from which their contents are directly released into the lumen (Figure 5f). Together with large goblet cells filled with secretory vesicles and electron-lucent nonsensory cells, slender dendrites bearing abundant cilia are observed in the central areas of the lamellae (Figure 5g).

### 3.3. Immunohistochemical (IHC) and Lectin-Histochemical (Lectin-HC) and Analyses

Although histological analysis provides valuable insights into cellular morphology, it does not allow clear discrimination of functional differences among cell subpopulations. Therefore, we present here the results of lectin-histochemical (Lectin-HC) and immunohistochemical (IHC) studies, employing lectins and primary antibodies widely used in olfactory research.

#### 3.3.1. IHC and Lectin-HC on Premetamorphic Larvae

The histological study provides detailed insight into the morphology of the olfactory organs during the first days of life, revealing marked structural differences when compared with more advanced developmental stages. To further characterize the neurochemical profile of the OE within the OF, we present the results of Lectin-HC labelling and IHC analyses.

**Figure 6:**
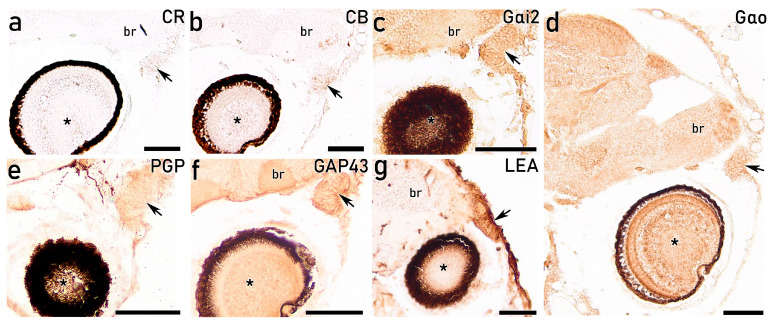
IHC and lectin-HC study of the olfactory fossae (OFs) on premetamorphic larvae (5 dph). (**a**) Anti-CR discretely stains some narrow cell processes. (**b**) Similarly, anti-CB barely produces a discrete immunoreaction in the OF. (**c**) Anti-Gαi2 generates an intense and generalized staining of the olfactory epithelium (OE) that extends into the brain (br). (**d**) Anti-Gαo produces a similar staining of the OE and the br. (**e**) Anti-PGP generates a slight staining of the OE. (**f**) Similarly to the staining with G-proteins (C and D), anti-GAP43 intensely stains the OE and the br. (**g**) HC labelling with the lectin LEA marks the OE intensely, producing a more intense labelling at the apical region. The arrows (**a**–**g**) indicate the olfactory epithelium, and an asterisk indicates the eye (**a**–**g**). br, brain. Scale bars: 50 μm (**a**–**g**).

The IHC study employing the calcium-binding proteins anti-calretinin (anti-CR) and anti-calbindin (anti-CB) barely reveals any immunoreaction on the OFs at the premetamorphic stage (Figure 6a,b). In contrast, the IHC study with the G-protein subunits Gαi2 and Gαo, key components of the olfactory signal transduction cascade, produces an intense immunoreactivity along the entire OE surface, continuing at the level of the brain (Figure 6c,d). The OE shows a diffuse immunopositivity with the antibody against PGP (Figure 6e). GAP43 produces strong immunoreactivity in the OE, comparable to that observed with antibodies against G-proteins (Figure 6f). Finally, lectin LEA generates a strong labelling within the OE, more pronounced at the luminal surface (Figure 6g). Overall, the distribution pattern of the employed markers supports the presence of functional sensory activity at this early developmental stage. However, these results do not allow reliable discrimination between cell subtypes using IHC and lectin-HC at this life stage.

#### 3.3.2. Lectin-HC on Fry and Juveniles

**Figure 7:**
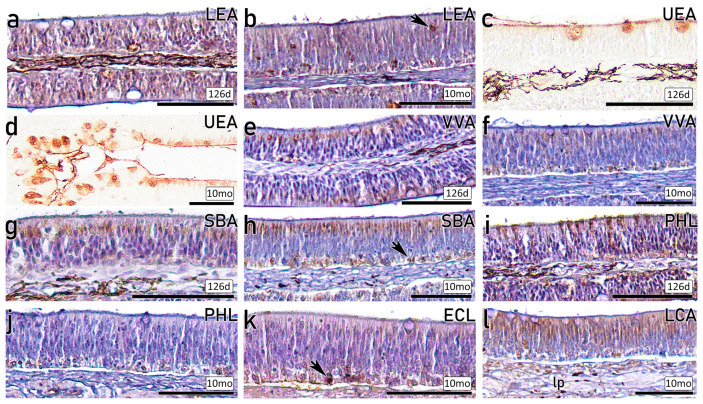
Lectin-HC study of the olfactory epithelium (OE) on fry of 126 dph (**a**,**c**,**e**,**g**,**i**) and juveniles of 10-month-old (10 mo) (**b**,**d**,**f**,**h,j**–**l**). (**a**,**b**,**e**–**l**) show the lectin-labelling after counterstaining with haematoxylin. (**a**) LEA shows labelling across the entire OE surface. (**b**) Similarly, LEA generates labelling across the OE, but more concentrated in the basal region and isolated globose cells in apical positions (arrow). (**c**,**d**) UEA staining of the goblet cells and cilia at the luminal surface on a 126 dph (**c**) and 10 mo (**d**) individuals. The OE shows no labelling. (**e**) Apical labelling of the OE with VVA. (**f**) VVA labelling is concentrated on the apical and most basal regions of the OE. (**g**,**h**) Similarly, the apical and basal regions of the OE are labelled with SBA on a 126 dph (**g**) and 10 mo (**h**), where basal rounded cells are intensely stained (arrow). (**i**) PHL staining on cilia in the lumen and apical cells projecting basally. (**j**) In contrast, PHL staining on a 10 mo juvenile generates a slight labelling at the basal region of the OE. (**k**) ECL strongly stains basal rounded cells (arrow). (**l**) LCA strongly labels the apical and central regions of the OE. Additionally, LCA generates certain staining within the lamina propria (lp). lp, lamina propria. Scale bars: 50 μm (**a**–**l**).

Lectin labelling produces distinct patterns across the OE of fry and juvenile individuals. Hematoxylin counterstaining was employed to facilitate the examination of different cell subpopulations. The lectin LEA produces labelling across the entire OE on a 126 dph (Figure 7a) and 10 mo (Figure 7b), although more concentrated in the basal layer and isolated apical globose cells. In contrast, lectin UEA only stains the goblet cells and the cilia of the luminal surface, while the OE remains negative (Figure 7c,d). A different labelling pattern is observed with the lectin VVA, which produces strong staining concentrated on the apical region on a 126 dph individual (Figure 7e), together with labelling of the basal region on a 10 mo individual (Figure 7f). Lectin SBA generates a similar labelling of the apical region and basal cells of the OE on a 126 dph (Figure 7g) and 10 mo (Figure 7h). Lectin PHL produces a distinct labelling across the different developmental stages, generating strong labelling of the cilia and apical cells on a 126 dph (Figure 7i), whereas only the basal region is stained on a 10 mo (Figure 7j). Lectin ECL allows the discrimination of strongly stained, rounded basal cells (Figure 7k). Finally, the epithelial layers exhibit a distinctive pattern with intense staining in the apical and central areas, whereas the basal region remains negative with lectin LCA (Figure 7l). Moreover, lectin LCA produces certain labelling within the lamina propria.

#### 3.3.3. Lectin-HC on Adults

**Figure 8:**
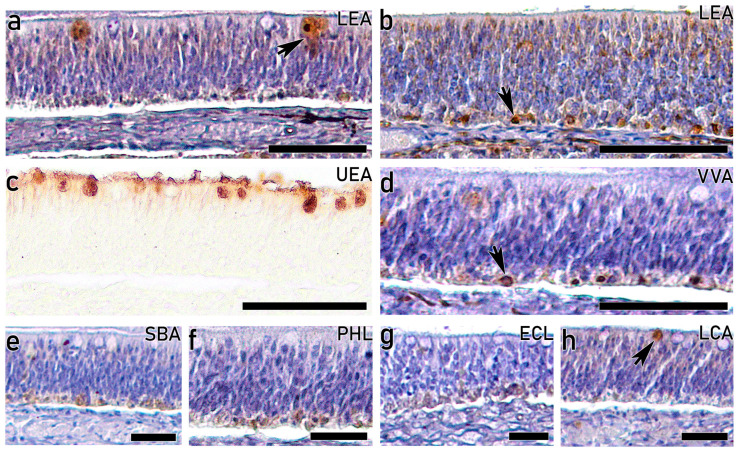
Lectin-HC study of the adult olfactory epithelium (OE). (**a**,**b**,**d**–**h**) show the staining of a lectin after counterstaining with haematoxylin. (**a**) LEA staining of the upper olfactory rosette shows a discrete labelling in the basal region of the OE, together with large, strongly stained, isolated apical deposits (arrow). (**b**) LEA staining of the lower olfactory rosette, showing strongly stained basal rounded cells (arrow), while less intensely labelled deposits are distributed throughout the apical region of the OE. (**c**) UEA staining shows strong positivity in rounded goblet cells and the luminal surface, while the OE is negative. In addition, the ciliated luminal surface displays intense immunoreactivity. (**d**) VVA staining of basal rounded cells (arrow) and isolated deposits across the OE. (**e**) SBA strongly stains the basal region. (**f**) Similarly to SBA, the staining with PHL shows strong staining of the most basal region of the OE, where rounded cells can be distinguished. (**g**) ECL produces a diffuse staining of the basal region of the OE. (**h**) LCA shows slight staining of the apical region of the OE, with isolated, rounded, strongly stained cells (arrow). Scale bars: 50 μm (**a**–**d**); 25 μm (**e**–**h**).

In accordance with lectin labelling on fry and juvenile stages, it enables the discrimination of cell subpopulations mostly located at the basal regions of adult individuals. Particularly, lectin LEA stains the basal region of the OE in both the upper (Figure 8a) and lower (Figure 8b) olfactory rosettes, together with large intensely stained apical deposits. As observed on fry and juveniles, lectin UEA strongly labels apical goblet cells and the luminal surface, while the OE remains negative (Figure 8c). The basal zone of the OE concentrates the labelling with lectins VVA (Figure 8d), SBA (Figure 8e), PHL (Figure 8f), ECL (Figure 8g) and LCA (Figure 8h). Furthermore, isolated deposits are stained with VVA, together with apical rounded cells with lectin LCA.

#### 3.3.4. IHC Study of the OE on Fry and Juveniles with Antibodies Against Calcium-Binding Proteins

**Figure 9:**
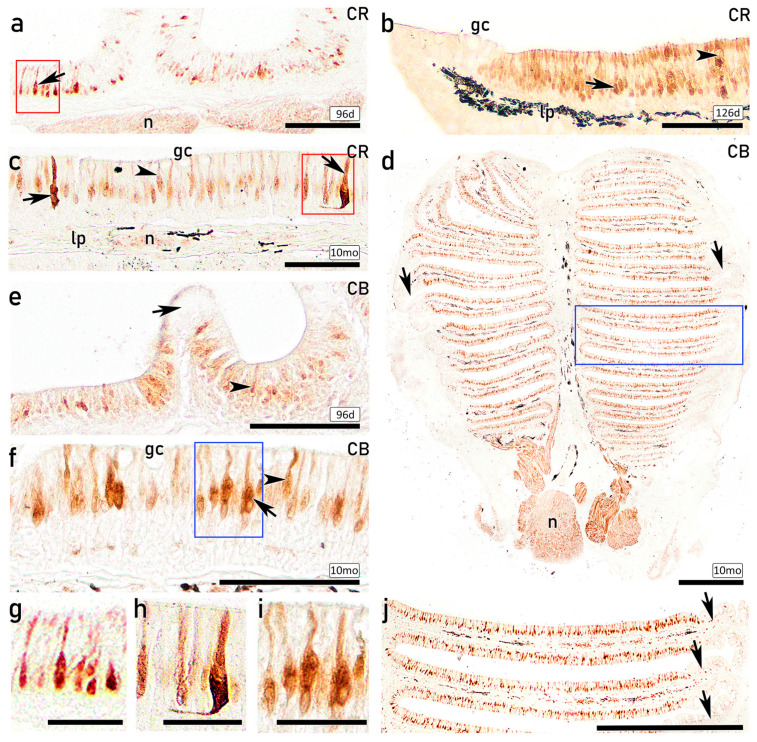
IHC-study of the olfactory epithelium (OE) in fry of 96 dph (**a**, e.g.) and 126 dph (**b**), and 10-month-old (10 mo) juveniles (**c**,**d**,**f**,**h**–**j**) using antibodies against calcium-binding proteins. (**a**) Anti-CR staining shows central elongated cell bodies projecting slender dendrites into the lumen (arrow). Basal to the OE, nerve bundles are immunopositive (n). (**b**) Anti-CR strongly immunostains the abundant elongated cells projecting narrow dendrites (arrow). Additionally, isolated oval cell bodies located apically show immunoreactivity (arrowhead). Abundant melanin deposits are observed in the lamina propria (lp). The peripheral region of the lamella is negative for anti-CR, including the goblet cells (gc). (**c**) Anti-CR staining reveals elongated, strongly immunopositive ciliated sensory cells projecting dendrites (arrows). Additionally, microvillous sensory cells with apical oval bodies and shorter projections are immunopositive (arrowhead). Gc are negative. The nerve bundles (n) in lp are immunopositive. (**d**) Complete horizontal section of the upper olfactory rosette of a 10 mo juvenile stained with anti-CB. This section clearly shows the boundaries between the sensory immunopositive epithelium in the central and inner regions of the lamella and the nonsensory negative epithelium in the peripheral region (arrows). Within the sensory region, abundant cells are anti-CB positive, together with strong immunoreactivity of the nerve bundles (n). (**e**) Anti-CB produces abundant immunopositive elongated cells projecting narrow dendrites to the lumen (arrowhead). The peripheral region of the lamella shows no immunoreactivity (arrow). (**f**) Ciliated sensory cells located centrally and projecting slender dendrites that reach the lumen are immunolabelled with anti-CB (arrow). Another subpopulation consists of positive cells with more apically located nuclei and shorter projections (arrowhead). The apical gc shows no immunoreactivity. (**g**) Higher magnification of the inset in (**a**) reveals the neuronal morphology. (**h**) Inset from (**c**) of the ciliated sensory cells strongly immunolabels with anti-CR. (**i**) Higher magnification of the inset in (**f**) shows the morphology of the immunopositive sensory neurons. (**j**) Higher magnification of the inset in (**d**) shows distinct epithelium regions, including a sensory region with abundant immunopositive cells with anti-CB and a negative nonsensory epithelium (arrows). n, nerve bundles; lp, lamina propria; gc, goblet cells. Scale bars: 50 μm (**a**–**c**,**e**,**f**); 500 μm (**d**,**j**); 20 μm (**g**,**h**,**i**).

Antibodies against calcium-binding proteins enable the discrimination of distinct subpopulations of OSNs across different developmental stages. IHC studies performed in fry (96 dph and 126 dph) and 10 mo juveniles using anti-CR and anti-CB revealed similar labelling patterns, with immunopositive OSNs located in the central and apical regions of the OE, and no immunoreactivity detected in the most basal part of the OE. Specifically, anti-CR strongly labels ciliated OSNs (cOSNs) characterized by elongated cell bodies projecting slender dendrites into the lumen as early as the 96 dph stage (Figure 9a), with an increased number of immunopositive cells observed in the 126 dph individuals (Figure 9b). Additionally, a population of microvillous OSNs (mOSNs) displaying oval cell bodies and shorter apical projections shows CR immunoreactivity in the 126 dph and the 10 mo individuals (Figure 9b,c). In the 10 mo juvenile, a distinct OSN subpopulation with large, broad cell bodies located in the central position and projecting thick dendrites can be distinguished (Figure 9c). Across all examined developmental stages, nerve bundles within the lamina propria exhibit CR immunoreactivity, whereas the peripheral region of the lamellae and secretory goblet cells across the entire OE remain immunonegative. Interestingly, immunolabelling of the entire olfactory rosette of 10 mo juvenile with anti-CB clearly differentiates the immunopositive sensory OE from the immunonegative nonsensory OE (Figure 9d). Consequently, CB-immunopositive OSNs are distributed across the entire epithelial surface, except for the peripheral region, which lacks immunoreactivity. Anti-CB immunolabels cOSNs located in deeper layers and projecting slender dendrites in both the 96 dph and the 10 mo individuals (Figure 9e,f). Additionally, a distinct subpopulation of more apically positioned mOSNs, displaying shorter dendrites, also exhibits CB immunoreactivity (Figure 9f). Different morphologies of immunopositive OSNs are observed with anti-CR (Figure 9g,h) and anti-CB (Figure 9i). The limits between the immunopositive sensory OE and immunonegative nonsensory epithelium are visible with anti-CB (Figure 9d,j).

#### 3.3.5. IHC Study of the OE on Adults with Antibodies Against Calcium-Binding Proteins

**Figure 10:**
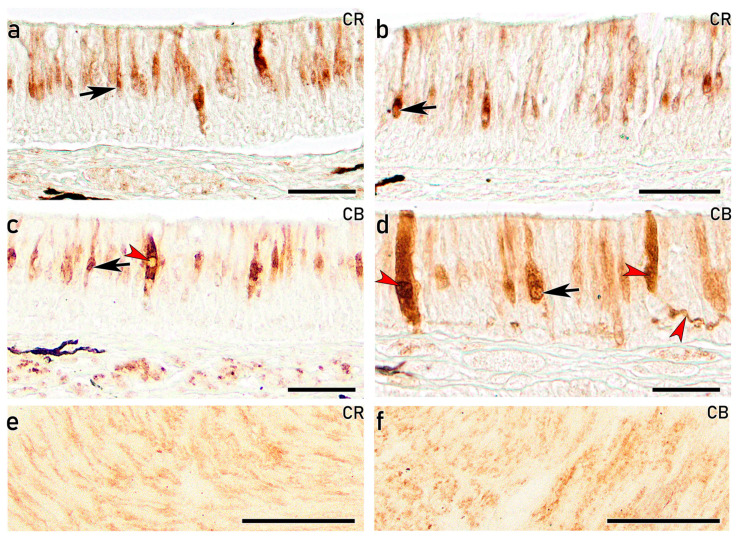
IHC study of the olfactory epithelium (OE) on the upper (**a**,**c**,**e**) and lower (**b**,**d**,**f**) olfactory rosettes of adults using antibodies against calcium-binding proteins. (**a**,**b**) Anti-CR produces a similar pattern in the upper (**a**) and lower (**b**) OE, immunolabelling ciliated olfactory sensory neurons (cONSs) displaying cell bodies in central positions with slender projections into the lumen (arrows). The basal part of the OE is negative. (**c**,**d**) Similarly, strong immunoreactivity is observed in the upper (**c**) and lower (**d**) OE with anti-CB, showing strong immunolabelling of cells with slender projections (arrows) and large cells with wide processes spanning the entire thickness of the epithelium (red arrowheads), both concordant with cOSNs. The basal region is immunonegative with anti-CB. (**e**,**f**) The nerve bundles are immunopositive for both anti-CR (**e**) and anti-CB (**f**). Scale bars: 25 μm (**a**–**f**).

In the adult OE, IHC study using the calcium-binding protein anti-CR reveals a pattern similar to that observed in earlier developmental stages (Figure 9). A subpopulation of cOSNs, centrally located and projecting slender and large dendrites, showed strong immunoreactivity in both the upper (Figure 10a) and the lower olfactory rosette (Figure 10b). These cOSNs are also immunolabelled with anti-CB in both the upper (Figure 10c) and lower (Figure 10d) olfactory rosettes. Furthermore, another subpopulation of cOSNs displaying wide cell bodies and projections occupying the entire epithelial surface shows strong immunoreactivity (Figure 10c,d). The nerve bundles are immunopositive with both anti-CR and anti-CB (Figure 10e,f). As observed in earlier developmental stages with these markers, the most basal part of the OE remains negative.

#### 3.3.6. IHC Study of the OE on Fry and Juveniles with Antibodies Against G-Proteins

**Figure 11:**
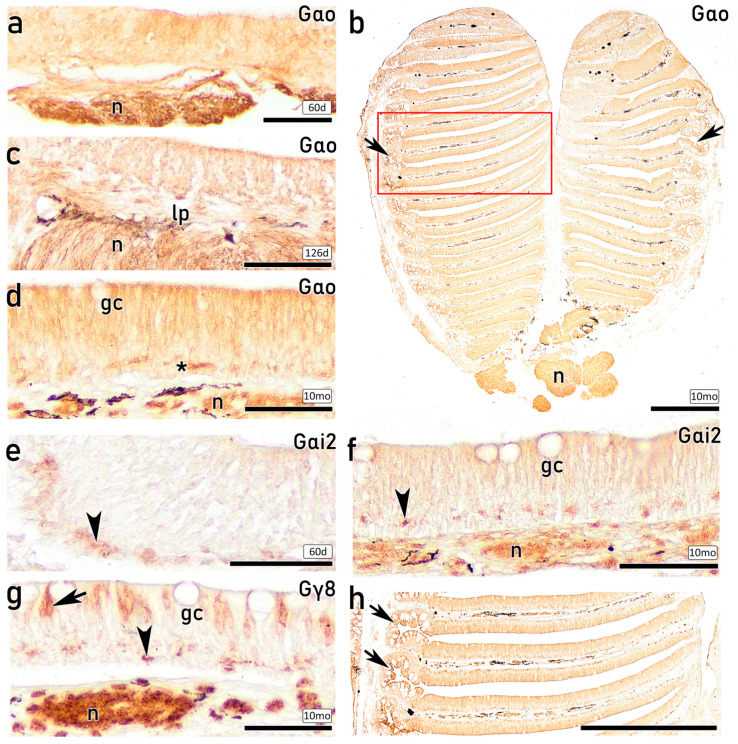
IHC study of the olfactory epithelium (OE) 60 dph (**a**,**e**) and 126 dph (**c**) fry and 10-month-old (10 mo) juveniles (**b**,**d**,**f**–**h**) using antibodies against G-proteins. (**a**,**c**) General immunolabelling of the OE with anti-Gαo on 60 dph (**a**) and 126 dph (**c**) fry, including strong immunoreactivity in the nerve bundles (n) within the lamina propria (lp). (**b**) Anti-Gαo immunolabelling of a horizontal section through the complete olfactory rosette of a 10-month-old, showing diffuse labelling of the OE along the entire lamellae, including the peripheral nonsensory regions (arrows) and the nerve bundles (n). (**d**) Uniform anti-Gαo immunostaining of the OE, along with scattered strongly stained basal areas (asterisk) and nerve bundles (n) in the lamina propria, where melanin deposits are also visible. Goblet cells (gc) are immunonegative. (**e**) Anti-Gαi2 showing immunostaining restricted to basal areas of the OE (arrowhead). (**f**) Anti-Gαi2 produces immunolabeling throughout the OE with stronger immunoreactivity in basal regions (arrowhead) and in the nerve bundles (n). Gc are immunonegative. (**g**) Anti-Gγ8 shows strong labelling of apically located OSNs reminiscent of mOSNs, showing oval cell bodies and short processes (arrow). Gc are immunonegative. In the basal region of the OE, discrete strongly stained areas are also observed (arrowhead). Nerve bundles (n) in the lamina propria are strongly immunostained. (**h**) Higher magnification of the inset in (**b**) shows diffuse immunostaining with anti-Gαo in both the sensory and nonsensory (arrows) parts of the lamellae. n, nerve bundles; lp, lamina propria; gc, goblet cells. Scale bars: 25 μm (**a**,**e**); 500 μm (**b**,**h**); 50 μm (**c**,**d**,**f**,**g**).

The use of different antibodies against G-protein subunits produces diffuse immunoreactivity throughout the OE, with stronger intensity in basal regions. In contrast to the immunolabelling observed for calcium-binding proteins, the peripheral non-sensory region also showed immunoreactivity for antibodies against G-proteins. Although the diffuse immunolabelling across the OE makes it challenging to discriminate between different OSN subpopulations, the olfactory nerve bundles within the lamina propria are immunopositive for the G-protein markers used, indicating positive labelling of OSNs. In contrast, the goblet secretory cells are immunonegative. Diffuse anti-Gαo immunoreactivity is observed in both 60 dph (Figure 11a) and 126 dph (Figure 11c) fry. The immunostaining of the entire OE with anti-Gαo is shown on a section of the complete olfactory rosette of a 10 mo juvenile (Figure 11b), displaying a similar pattern with occasionally stronger immunolabelling at the basal position (Figure 11d). Anti-Gαi2 produces weak immunolabelling of the OE in 60 dph individuals, with greater intensity in basal regions (Figure 11e). In 10 mo juvenile (Figure 11f), anti- Gαi2 produces stronger immunolabelling in the OE, again more pronounced basally. Interestingly, anti-Gγ8 immunostains a subpopulation of apical mOSNs characterized by oval cell bodies projecting narrow and short dendrites, together with stronger immunolabelling in basal regions and in the olfactory nerve bundles within the lamina propria (Figure 11g).

#### 3.3.7. IHC Study of the OE on Adults with Antibodies Against G-Proteins

**Figure 12:**
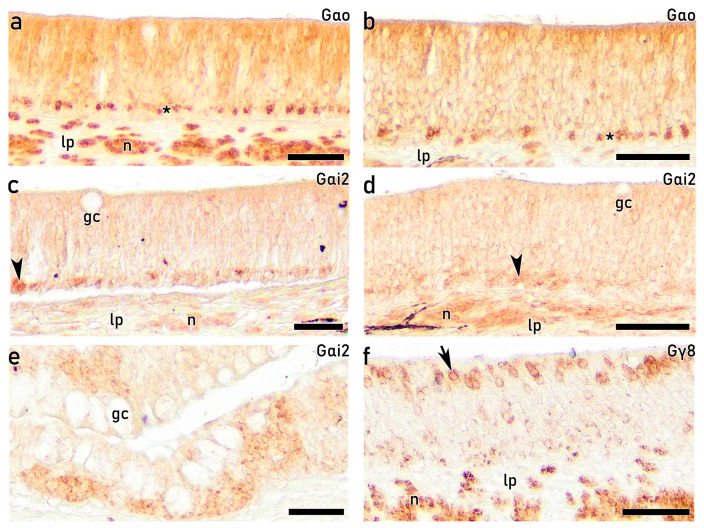
IHC study of the olfactory epithelium (OE) in the upper (**a**,**c**,**e**,**f**) and lower (**b**,**d**) olfactory rosettes of adult individuals using antibodies against G-proteins. (**a**,**b**) Anti-Gαo produces strong immunostaining in the OE of both the upper (**a**) and lower (**b**) rosette, including strongly stained rounded cells in the basal position (asterisks). In the lamina propria (lp), nerve bundles (n) are strongly stained. (**c**,**d**) Anti-Gαi2 produces generalized staining of the OE in both the upper (**c**) and lower (**d**) rosette, more intense in the basal position (arrowheads). In lp, nerve bundles (n) are also immunopositive, whereas goblet cells (gc) are immunonegative. (**e**) Peripheral region of a lamella showing the nonsensory OE, with abundant immunonegative gc and strong anti-Gαi2 staining in deeper layers. (**f**) Anti-Gγ8 immunolabels a subpopulation of short and oval OSNs in apical positions (arrow). Nerve bundles (n) in the lp are also labelled. n, nerve bundles; lp, lamina propria; gc, goblet cells. Scale bars: 25 μm (**a**–**f**).

In the adult OE, in accordance with that observed in fry and juveniles, IHC study using antibodies against G-proteins reveals a diffuse immunolabelling pattern, making it challenging to discriminate between cell subtypes. This pattern is observed with anti-Gαo, which shows diffuse labelling together with stronger staining of rounded cells located at basal regions in both the upper rosette (Figure 12a) and lower rosette (Figure 12b). Additionally, the olfactory nerve bundles are strongly stained in both rosettes. Anti-Gαi2 produces a similar pattern in both the upper rosette (Figure 12c) and lower rosette (Figure 12d). At the peripheral region of the lamellae, anti-Gαi2 shows no immunoreactivity in secretory goblet cells, while a strong immunoreaction is detected in deeper layers (Figure 12e). Notably, anti-Gγ8 markedly increases in intensity from the juvenile stage, immunolabelling a subpopulation of apical short and oval mOSNs, together with the nerve bundles at the lamina propria (Figure 12f). These cells morphologically match the apical, oval receptor-like cells indicated by the arrowhead in the H&E-stained section (Figure 3c).

#### 3.3.8. Additional IHC Markers on the OE of Fry, Juvenile and Adults

**Figure 13:**
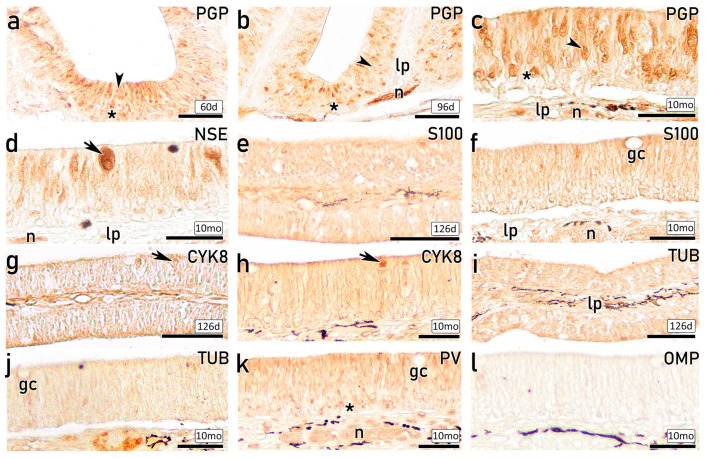
IHC study of the olfactory epithelium (OE) in in fry of 60 dph (**a**), 96 dph (**b**), 126 dph (**e**,**g**,**i**) and juveniles of 10-month-old (10 mo) (**c**,**d**,**f**,**h**,**j**–**l**). (**a**–**c**) Anti-PGP displays immunoreactivity in different cell populations throughout the OE, including rounded basal cells (asterisks in (**a**–**c**)), and spindle-shaped central and apical cells (arrowhead). Moreover, apical oval cells with short dendrites that resemble microvillous sensory neurons (mOSNs) are immunopositive (arrow in (**c**)). Nerve bundles (n) in the lamina propria (lp) are also immunopositive. (**d**) Anti-NSE slightly immunolabels the OE, discriminating globose cells bodies at the apical position, resembling crypt cells (CC) (arrow). In lp, nerve bundles (n) are immunopositive. (**e**) Anti-S100 reveals diffuse immunolabeling of the entire OE, discriminating globose cell bodies at the apical position resembling CC (arrow). (**f**) Similarly, anti-S100 stains the OE, except for gc. (**g**,**h**) Anti-CYK8 diffusely labels the OE in a 126 dph (**g**) and 10 mo (**h**). A subpopulation of globose apical cells resembling CCs shows strong immunoreactivity (arrows). (**i**,**j**) Anti-TUB produces generalized immunolabeling of the OE in 126 dph (**i**) and 10 mo (**j**), with immunonegative gc. (**k**) Anti-PV shows diffuse staining of the OE. Additionally, the basal epithelial region shows stronger immunoreactivity of small and rounded cells (asterisk). Gc are negative, and nerve bundles (n) are immunopositive. (**l**) Anti-OMP produces no immunoreactivity on a 10 mo individual. n, nerve bundles; lp, lamina propria; gc, goblet cell; CC, crypt cell. Scale bars: 25 μm (**a**,**b**); 50 μm (**c**–**l**).

Other markers employed at the IHC study allow for better discrimination among various cell morphologies across the entire OE from early developmental stages, reflecting a highly developed OE in this species. Anti-PGP immunolabels abundant OSNs located in central and apical positions on 60 dph (Figure 13a) and 96 dph (Figure 13b) fry, and 10 mo juveniles (Figure 13c). Furthermore, rounded cells in more basal positions show immunoreactivity, together with nerve bundles in the lamina propria. The antibody against NSE produces weak immunolabelling across the entire OE but allows a discrimination of a subpopulation of globose cell bodies and basal nuclei located in apical positions consistent with CCs (Figure 13d). Despite the diffuse labelling throughout the OE with anti-S100, a weak immunolabelled subpopulation of cells resembling CCs is observed in fry of 126 dph (Figure 13e). In contrast, such cells were not clearly identifiable in a 10 mo individual examined here (Figure 13f). Furthermore, these CCs, although with lower intensity, are immunolabelled with anti-CYK8, which marks both sensory and nonsensory supporting cells throughout the entire OE (Figure 13g,h). Similarly to anti-S100, the IHC study with anti-TUB (Figure 13i,j) and anti-PV (Figure 13k) produces a diffuse immunolabelling of the OE in juvenile individuals. Notably, the anti-PV marker allows discrimination of a subpopulation of rounded basal cells, together with positive nerve bundles (Figure 13k). Finally, the OE shows no immunoreactivity with anti-OMP (Figure 13l).

**Figure 14:**
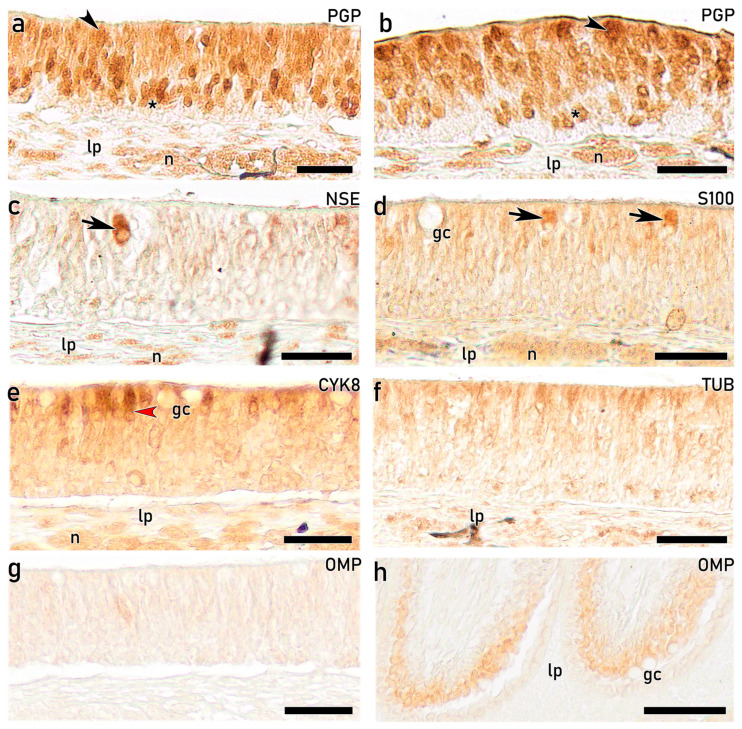
IHC study of the olfactory epithelium (OE) in adults. (**a**,**b**) Anti-PGP immunolabeling of the upper (**a**) and lower (**b**) olfactory rosette reveals a general immunostaining of the OE, with positive cells distributed along the OE, including basal rounded cells (asterisks) and apical cells ranging from oval to more elongated shapes (arrowheads). The nerve bundles (n) in the lamina propria (lp) are also immunopositive, whereas the most basal part of the OE lacks immunopositive cells. (**c**) Isolated globose cells consistent with crypt cells (CCs) (arrow) are strongly labelled with anti-NSE (arrow). Nerve bundles (n) in the LP are also positive. (**d**) Anti-S100 similarly labels crypt cells (arrows), although the OE shows intense diffuse staining. Goblet cells (gc) are immunonegative. (**e**) Anti-CYK8 diffusely labels the OE, with stronger immunoreactivity in apical cells with wide cell bodies (red arrowhead), and negative gc. The nerve bundles (n) in lp are positive. (**f**) Anti-TUB show immunoreactivity throughout the OE, more intense in apical regions. Gc are negative. (**g**) No immunoreactivity is detected in the sensory OE with anti-OMP. (**h**) Peripheral area of a lamella showing strong anti-OMP labelling in deeper epithelial layers. n, nerve bundles; lp, lamina propria; gc, goblet cell; CC, crypt cell. Scale bars: 25 μm (**a**–**h**).

The same markers applied to the adult OE produce similar results to those described for fry and juvenile individuals. Notably, anti-PGP provides the most detailed insights in terms of cell discrimination and distribution across the OE in both the upper (Figure 14a) and lower (Figure 14b) rosettes, except at the most basal layer, which remains immunonegative. Immunopositive OSNs include rounded cells located in central positions, as well as oval and more elongated cells at apical positions resembling mOSNs. Olfactory nerve bundles in the lamina propria are strongly labelled. As observed in juveniles, anti-NSE specifically immunolabels CCs (Figure 14c). Furthermore, these CCs show immunoreactivity with anti-S100, together with diffuse immunolabelling of the OE and positive nerve bundles (Figure 14d). Anti-CYK8 diffusely labels the OE and nerve bundles, allowing discrimination of a subpopulation of apical nonsensory supporting cells (Figure 14e). In contrast, IHC with anti-TUB (Figure 14f) produces generalized immunolabelling of the OE, preventing discrimination among different cell subtypes. Finally, no immunoreactivity with anti-OMP is observed in the sensory OE (Figure 14g), whereas the nonsensory OE at the peripheral area of the lamellae shows strong immunolabel in deeper layers (Figure 14h).

## 4. Discussion

*S. senegalensis* is a highly promising aquaculture species in Europe, for which substantial efforts are still ongoing to establish an efficient breeding system under captive conditions. Over recent years, research has integrated genomic, physiological and behavioural approaches aimed at elucidating the reproductive dysfunction that continues to constrain its expansion [29,32,45,46,47]. In this context, altered chemoperception abilities, hypothetically originating from hatchery conditions during early life stages, have been hypothesized as a potential key factor underlying impaired reproductive behaviour in captive-bred males [16,27,29]. The influence of chemoperception through olfaction in fish reproduction is widely known [11,31,48]. Moreover, recent genomic studies have revealed a particularly developed olfactory system in *S. senegalensis*, including an expanded olfactory receptor gene repertoire compared to other flatfish [33,35,36]. Despite the growing molecular and functional evidence, the morphological and neurochemical characterization of the olfactory organs in this species remains limited and largely restricted to basic histological descriptions. In this context, this study provides a comprehensive histological, ultrastructural, lectin-HC and IHC characterization of the olfactory organs of *S. senegalensis*, establishing a structural and neurochemical framework that supports and complements previous functional and transcriptomic findings, ultimately contributing to a better understanding of the molecular basis of olfaction in the species.

### 4.1. Olfactory Rosettes Morphology, Lamellae Arrangement and Epithelial Organization

Our morphological examination shows a well-developed olfactory epithelium (OE) from early life stages. The incipient organization observed in the olfactory fossae (OF) of premetamorphic larvae transforms into a conserved organizational pattern of the olfactory rosettes of *S. senegalensis* across all stages analyzed, consistent with previous reports in flatfish species [8,25,26]. After metamorphosis, only minor morphological changes were observed, including an increased number of lamellae and the overall enlargement of the olfactory rosettes in more advanced stages, as reported in other teleosts [10,49]. Although different lamellar arrangements have been described among flatfish species, *S. senegalensis* exhibits a perfectly parallel lamellar organization, especially in the upper rosette, in contrast to the more radial configuration reported in turbot [26]. *S. senegalensis* shows a multilamellar olfactory rosette, like most teleosts, although the absence of lamellae has been observed in species displaying similar OE development [50,51].

In adults, the examination of the upper and lower olfactory rosettes revealed a bigger upper olfactory rosette with a greater number of lamellae, in accordance with previous observations in this species [34]. Although functional asymmetry between the upper and lower rosettes has been reported [52], no histological or neurochemical disparities were detected in the present study between them under standard captive conditions. In contrast, histological differences have been reported after water acidification [34]. Future studies incorporating sex-related comparisons and morphometric analyses of the olfactory organ may provide further insights, particularly in the context of reproductive dysfunction in this species, as recent studies have reported sexual dimorphism in olfactory organ size and lamellar number [53].

Two distinct epithelial patterns were identified across the lamellae: (i) a pseudostratified columnar sensory epithelium located in their central and inner parts and (ii) a stratified nonsensory epithelium occupying the peripheral areas. The epithelial differentiation observed in the histological analysis was confirmed by immunohistochemical labelling with antibodies against calcium-binding proteins (anti-CR and anti-CB), as well as by TEM observation, which confirmed the presence of two distinct epithelial organizations. A similar epithelial arrangement has been previously reported in other flatfish species [54] and zebrafish [43]. In contrast, a more interspersed epithelial arrangement along the lamellar surface has been reported in other teleosts [26,55,56]. The OE relies on a basal lamina above the underlying lamina propria, where axons projecting from the OSNs converge to ultimately form the olfactory nerve. While this structure was discernible in routine histological sections, ultrastructural analysis revealed a thick and well-defined basal lamina in *S. senegalensis*, consistent with previous descriptions in turbot [26].

### 4.2. Morphological and Neurochemical Features of the Olfactory Organs on Premetamorphic Larvae

The olfactory organs of premetamorphic larvae, referred to as olfactory fossae (OFs), consisted of a flat epithelial layer lacking lamella, consistent with previous observations in *S. senegalensis* [37] and other flatfish [25,26]. The OFs were located in the anterodorsal region of the head, displaying a spatial arrangement comparable to that observed in bilaterally symmetrical teleost species [43]. After metamorphosis, the organ acquires a multilamellar morphology that persists throughout subsequent developmental stages. Despite the marked structural disparities between the OFs and the multilamellar olfactory rosettes, the OE in premetamorphic larvae already exhibits a dense cellular epithelium, encompassing diverse cell morphologies that persist in the adults’ OE. As previously observed in premetamorphic turbot [25,26], strong immunoreactivity for neuronal markers, such as anti-Gαi2, anti-Gαo, anti-PGP and anti-GAP43, was observed, together with weaker immunoreactivity for anti-CR and anti-CB. These findings support that functionally differentiated OSNs are already present within the OFs of *S. senegalensis* at premetamorphic stages, consistent with the observations in other teleost species of the emergence of sensory cells after the first day of hatching [57].

### 4.3. Morphological and Neurochemical Characterization of the Olfactory Sensory Epithelium (OE)

The limited information on *S. senegalensis*, together with the small cell size, high cellular density and tightly packed cells, makes the characterization of the olfactory epithelium particularly challenging. However, the integrated approach conducted in the present study enabled the discrimination of cell subtypes based on their morphology, topological distribution within the epithelium, and specific immunoreactive patterns. As widely described across fish, the sensory OE is composed of OSNs, supporting cells, mucus-secreting goblet cells and basal cells [10,19]. Among OSNs, ciliated OSNs (cOSNs), microvillous OSNs (mOSNs) and crypt cells (CCs) were observed in *S. senegalensis*, consistent with previous reports in teleost [43,55,58]. However, no kappe and pear OSNs subtypes were detected in the present study, which have only been described in zebrafish [59,60].

In line with this cellular composition, the central and inner parts of the lamellae exhibited a pseudostratified epithelium composed of OSNs and supporting cells of varying morphologies, together with basal rounded cells and apical goblet cells. These observations coincide with those previously reported in the species [32,34]. Although routine histological techniques have been previously conducted on the OE of *S. senegalensis*, to our knowledge, a detailed characterization of distinct cellular subtypes integrating morphological, spatial, and neurochemical features has not been reported. The ultrastructural and IHC analyses performed in the present study provide new insights into the cellular organization and neurochemical profile of the OE in this species.

In the deeper layers of the OE, cOSNs were abundant, displaying the characteristic spindle-shaped cell bodies and elongated apical projections toward the luminal surface, where they terminated in cilia. These morphological features are consistent with the well-established morphology of cOSNs in teleost [13,14]. Ultrastructural analysis confirmed the presence of large cOSNs, which occasionally spanned the entire thickness of the OE. These cOSNs displayed electron-dense bodies, nuclei with granular heterochromatin, and slender apical projections bearing cilia into the lumen. Such ultrastructural observations represent hallmark features of mature cOSNs and align with previous descriptions in several teleost species [26,42,55,56,61].

Located more apically and related to cOSNs, mOSNs were identified by their oval-shaped cell bodies and shorter apical dendrites bearing microvilli. This morphology corresponds to that widely reported in other fish [10,62,63]. TEM confirmed these observations, revealing electron-dense mOSNs with oval bodies, rounded nuclei, and shorter apical projections terminating in microvilli. The relative position, morphology, and ultrastructural features of both neuronal populations observed in the present study agree with previous histological and ultrastructural descriptions of teleost OE, supporting the distinction between cOSN and mOSN subpopulations [26,42,43,55,56].

IHC analyses further confirmed the neuronal identity and differentiation status of both cOSNs and mOSNs. From 96 dph onward, both populations exhibited clear immunoreactivity against CR, CB, and PGP, indicating advanced neuronal differentiation and functional maturation since early life stages. Calcium-binding proteins such as CR and CB have been widely associated with mature OSNs in teleost fishes and are implicated in calcium homeostasis and activity-dependent regulation [43,64,65,66]. Moreover, PGP, a well-established neuronal marker expressed in both differentiating and mature OSNs [41,67], enabled identification of abundant immunopositive OSNs with varying morphologies distributed in the upper three-quarters of the OE, in agreement with previous reports [68,69]. Thus, anti-PGP emerges as a robust marker for investigating OSNs subtypes in the *S. senegalensis* OE. In contrast, although anti-Gαo immunoreactivity has been previously associated with mOSNs linked to the mammalian vomeronasal receptors V2R [70], in the present study, it produced diffuse epithelial labelling without clear identification of individual mOSNs, despite strong staining of nerve bundles in the lamina propria. Notably, anti-Gγ8 immunolabelling was specifically associated with mOSNs, emerging as a specific immunomarker of mOSNs in *S. senegalensis*. Overall, the combined histological, ultrastructural, and IHC findings presented here are consistent with previous studies in teleost and confirm the presence of structurally and functionally differentiated OSN populations in the OE of *S. senegalensis*. Although these findings are supported by immunoreactivity, further validation of antibody specificity would be required to fully confirm marker expression in this species.

Crypt cells (CCs), another well-recognized neuronal subtype in teleost OE, were histologically identified in *S. senegalensis* from the 60 dph stage onward. These neurons displayed the characteristic globose bodies located in apical positions and basal nuclei, consistent with the typical morphology described for fish CCs [63,71]. In teleosts, CCs typically express a single V1R-related ORA gene, directly involved in pheromone detection [72,73,74]. Immunolabelling with anti-S100, a marker widely used for CCs identification in fish [64,66,75,76], produced widespread immunolabelling throughout the OE of *S. senegalensis*. Nevertheless, it enabled the clear recognition of intensely immunolabelled CCs, based on their distinctive morphology and apical localization. Additionally, immunoreactivity with anti-NSE and anti-CYK8 selectively labelled these CCs along *S. senegalensis* OE, further supporting their neuronal identity. The combined immunophenotypic profile observed here suggests a degree of species-specific antigen expression across teleost species, in line with previous proposals of functional heterogeneity within this neuronal subtype [76]. The consistent presence and relative abundance of CCs throughout juvenile and adult stages indicate their sustained role in the olfactory-mediated chemical communication in *S. senegalensis*.

In the basal region of the OE, adjacent to the basal lamina, small, rounded basal cells were consistently observed. Ultrastructural examination revealed nuclei with variable morphologies and chromatin condensation, consistent with progenitor cell populations described in teleost OE [10,77]. These characteristic basal cells constitute a proliferative population responsible for the continuous renewal and differentiation of OSNs, ensuring maintenance and functional integrity of the OE [14,19,78,79].

Notably, a basal subpopulation of rounded cells exhibited immunoreactivity for anti-Gαo, similar to that previously observed in turbot [26]. Immunoreactivity for anti-Gαi2 was likewise observed in the basal portion of the OE. These G-protein subunits are associated with vomeronasal-type receptor signalling pathways, particularly V1R- and V2R-related receptors, which play a central role in pheromone detection and chemosensory communication in vertebrates, including teleost and cartilaginous fishes such as sharks [70]. The presence of Gαo- and Gαi2-immunopositive basal cells is compatible with their identification as immature or differentiating OSNs, as G-protein subunits are known to be expressed during early stages of neuronal differentiation [14,19,80]. Furthermore, these basal rounded cells showed lectin-labelling, consistent with previous observations in other flatfish, in which glycoconjugate expression has been associated with neuronal differentiation and epithelial regeneration [26,68]. This early engagement of vomeronasal-related signalling components in basal progenitor populations may indicate that receptor-specific signalling pathways are established prior to full morphological maturation.

Overall, the IHC approach offered a clearer image for the discrimination of distinct cell subpopulations, supporting the marked complexity and heterogeneity of the OE from early developmental stages. In contrast, other immunomarkers failed to provide clear cellular discrimination. Notably, anti-OMP, a common marker for mature OSNs [81,82], did not produce detectable immunoreactivity in the present material. This absence of protein-level detection contrasts with previous transcriptomic analyses reporting the expression of the olfactory marker protein gene in the olfactory rosettes of *S. senegalensis* [33]. Further investigation using species-specific antibodies or single-cell or spatial transcriptomics would help clarify the temporal and functional dynamics of OMP expression in this flatfish.

The high proportion of OSNs observed throughout the OE of *S. senegalensis* is consistent with the expanded olfactory receptor repertoire reported for this species, comprising 455 olfactory receptor genes distributed among the four multigene families: OlfC, OR, ORA and TAAR [36]. Although the precise cellular expression patterns of these genes within the OE of *S. senegalensis* remain to be determined, previous studies in vertebrates, including teleosts, have characterized their distribution among distinct neuronal subtypes. Typically, OR and TAAR genes are predominantly expressed in cOSNs, whereas OlfC genes are mainly associated with mOSNs [14,19,80]. While some ORA genes have been linked to mOSNs, CCs are considered a specialized neuronal subtype for ORA gene expression [73,74]. Nevertheless, considerable interspecific variation exists in the size, composition, and functional organization of olfactory receptor repertoires among teleost, reflecting ecological pressures, behavioural strategies, and evolutionary diversification [2,83]. Therefore, species-specific functional and molecular studies are necessary to further elucidate the cellular expression patterns and physiological roles of olfactory receptor genes in *S. senegalensis*, ultimately contributing to a better understanding of its olfactory sensitivity and ecological adaptations.

### 4.4. Organization and Functional Features of the Nonsensory Olfactory Epithelium

The integrated histological, IHC and ultrastructural analyses performed in the present study revealed two clearly differentiated epithelial organizations throughout the olfactory lamellae, corresponding to sensory and nonsensory areas. Within the sensory area, OSNs were tightly surrounded by sustentacular nonsensory cells, forming a highly compact cellular arrangement that hindered the discrimination between cell subtypes by routine histology alone. Ultrastructural examination proved essential for resolving these cellular differences. Compared to OSNs, the sustentacular nonsensory cells were characterized by elongated, electron-lucent profiles extending apical processes bearing both cilia and microvilli into the lumen. These morphological features are consistent with their established role in providing mechanical support and contributing to the epithelial integrity [10,13].

Remarkably, the apical cytoplasm of sustentacular cells contained abundant electron-lucent secretory granules, indicative of active secretion into the luminal surface. Similar secretory granules have been previously described in other teleost species [55] and are thought to contribute to mucus production and epithelial protection [10]. Moreover, abundant secretory goblet cells densely packed with secretory vesicles were distributed along the lamella, particularly in the peripheral region. These goblet cells represent specialized secretory elements responsible for producing mucus that contributes to epithelial protection [84]. The peripheral lamellar regions featured nonsensory cells of varying morphologies and dispositions, supporting a highly complex epithelial organization, as confirmed by TEM, with abundant goblet cells and diverse nonsensory cell morphologies. Consistent with their secretory role, goblet cells lacked immunoreactivity for neuronal markers but showed strong lectin binding with UEA, supporting previous lectin-HC observations of glycoconjugate-rich mucus in teleost OE [26,68]. Additionally, electron-dense nonsensory cells bearing numerous apical microvilli were observed, further highlighting the marked cellular heterogeneity and functional complexity of the nonsensory OE. The structural diversification of the nonsensory epithelium reflects a dual function for protecting the sensory surface through mucus secretion and barrier/immune mechanisms, as well as for modulating the chemical microenvironment at the epithelial surface [85,86,87]. The latter is particularly relevant in benthic flatfish, which operate near the substrate and are continuously exposed to sediment-associated particles and chemical stimuli [88].

### 4.5. Lectin-HC Labelling of the Olfactory Epithelium (OE)

During the early larval stage (5 dph), lectin LEA generated a diffuse labelling, whereas fry, juvenile and adult stages exhibited stratified and region-specific labelling patterns. This transition suggests a progressive remodelling of the epithelial glycocalyx accompanying the structural and functional maturation of the OE following metamorphosis, a phenomenon previously described in developing teleost sensory epithelia and associated with epithelial differentiation and cellular specialization [68]. Among the lectins tested, UEA showed a distinctive labelling pattern, selectively marking goblet cells and the luminal surface, while sparing the sensory epithelium. This distribution supports the clear compartmentalization between sensory and nonsensory regions, highlighting the protective role of mucus [13].

In contrast, the concentrated labelling of the basal region of the OE with lectins LEA, ECL, SBA, VVA and PHL suggests a specific glycosylation profile associated with proliferative and differentiating cell populations near the basal lamina. These basal cells constitute the proliferative compartment of fish OE, continuously generating new OSNs, and changes in glycoconjugate expression have been linked to epithelial renewal and neuronal differentiation [77]. Moreover, differences in lectin binding patterns with VVA and PHL between 126 dph fry and 10 mo juvenile point to postmetamorphic epithelial remodelling and maturation of the luminal environment and mucus composition. Mucus changes through development from larvae to juvenile stages have previously been described in the skin of *S. senegalensis* [89] and in turbot [90]. These modifications in epithelial glycoconjugates are thought to influence odourant solubilization and epithelial protection [41]. Overall, the heterogeneous lectin binding profiles observed across the different life stages in *S. senegalensis* support the structural and functional complexity of the olfactory epithelium, supporting the multiple roles of the OE in epithelial maintenance, efficient odour detection and modulation of the chemical microenvironment [19,77].

## 5. Conclusions

The present study provides the first comprehensive characterization of the structural and neurochemical organization of the olfactory epithelium in *Solea senegalensis* across multiple life stages. Although the sample size was limited in some stages, the consistency of the observed patterns supports the reliability of the morphological characterization. The diversity of neuronal populations and epithelial components described here constitutes a morphological substrate compatible with the expanded olfactory receptor gene repertoire previously reported for this species. Notably, the presence of differentiated olfactory sensory neurons in premetamorphic stages indicates that functional chemosensory capability is established before the complete morphological maturation of the olfactory organ. In addition, the pronounced structural specialization observed in the nonsensory epithelium suggests adaptive mechanisms aimed at protecting the sensory surface and modulating the chemical microenvironment, particularly relevant for a benthic species constantly exposed to sediment-associated particles and dissolved compounds. Altogether, these findings significantly expand the current knowledge of the olfactory system in *S. senegalensis* and provide an important anatomical and neurochemical framework for future studies addressing olfactory function. Such information may contribute to a better understanding of the mechanisms underlying altered chemical communication and reproductive dysfunction observed in captive males of this species.

## Figures and Tables

**Table 1:** Detailed information on the antibodies used in the present study.

Antibody	Type	Species	Dilution	Supplier & Catalog No.	Immunogen
Anti-CR	Polyclonal	Rabbit	1:200	ProteinTech 12278-1-AP (Rosemont, IL, USA)	Recombinant human calretinin containing an N-terminal 6xHis tag
Anti-CB	Polyclonal	Rabbit	1:200	ProteinTech 14479-1-AP	Recombinant rat calbindin D-28k
Anti-GAP43	Polyclonal	Rabbit	1:200	ProteinTech 16971-1-AP	GAP43 fusion protein Ag9294
Anti-PGP	Polyclonal	Rabbit	1:200	ProteinTech 14730-1-AP	UCHL1/PGP9.5 fusion protein Ag6490
Anti-Gαo	Polyclonal	Rabbit	1:100	Santa Cruz SC-387 (Dallas, TX, USA)	Gαo subunit of the bovine GTP-binding protein
Anti-Gαi2	Polyclonal	Rabbit	1:100	ProteinTech 11136-1-AP	Peptide mapping within a highly divergent domain of rat Gαi2
Anti-Gγ8	Polyclonal	Rabbit	1:150	Cloud-Clone PAQ769Mu01 (Katy, TX, USA)	Recombinant Gγ8 expressed in *E. coli*
Anti-NSE	Polyclonal	Rabbit	1:150	Cloud-Clone Corp PAA537Mu01	Recombinant NSE expressed in *E. coli*
Anti-S100	Polyclonal	Rabbit	1:100	Cloud-Clone Corp PAA012Hu01	Recombinant S100 expressed in *E. coli*
Anti-CYK8	Polyclonal	Rabbit	1:100	ProteinTech 17514-1-AP	Recombinant peptide
Anti-TUB	Polyclonal	Rabbit	1:100	ProteinTech 10068-1-AP	Beta tubulin fusion protein
Anti-PV	Polyclonal	Rabbit	1:100	ProteinTech 26521-1-AP	Recombinant peptide
Anti-OMP	Monoclonal	Mouse	1:100	Santa Cruz SC-365818	Human OMP Amino Acid 1-163

**Table 2:** Detailed information on the lectins used in the present study.

Lectin	Abbreviation	Dilution (mg/mL)	Catalog No.	Preferred Sugar Specificity
*Lycopersicon esculentum* (tomato) lectin	LEA	1.0	Vector B-1175-1 (Vector Laboratories, Newark, CA, USA)	β-1,4 N-acetyl-glucosamine oligomers
*Ulex europaeus* agglutinin	UEA	2.0	Vector B-1065–2	α-Fucose
*Vicia villosa* agglutinin	VVA	2.0	Vector B-1235-2	N-acetyl-galactosamine
*Glycine max* (soy-bean) lectin	SBA	2.0	Vector B-1015-5	N-acetyl-galactosamine
*Phaseolus vulgaris* lectin	PHL	2.0	Vector B-1115-2	mannose residues and glucose
*Erythrina cristagalli* lectin	ECL	2.0	Vector B-1145-2	D-galactose and N-acetyl-galactosamine
*Lens culinaris* agglutinin	LCA	2.0	Vector B-1045-2	D-galactose residues

## Data Availability

The original contributions presented in this study are included in the article. Further inquiries can be directed to the corresponding author.

## Abbreviations

The following abbreviations are used in this manuscript:

OE	Olfactory epithelium
cOSN	Ciliated olfactory sensory neuron
mOSN	Microvillous olfactory sensory neuron
CC	Crypt cell
dph	Days post hatching
mo	Month-old
H&E	Hematoxylin and eosin
TEM	Transmission electron microscope
IHC	Immunohistochemistry
HC	Histochemistry
BSA	Bovine serum albumin
DAB	diaminobenzidine
ABC	Avidin-biotin-peroxidase
OF	Olfactory fossae
oc	Oral cavity
dg	Digestive tract
ot	Otic capsule
br	brain
n	Nerve bundles
gc	Goblet cell
bc	Basal cell
cr	Central raphe
lp	Lamina propria
nsc	Nonsensory cell
ci	cilia
mi	microvilli
sv	Secretory vesicles
CR	calretinin
CB	calbindin
GAP43	Growth associated protein 43
PGP	Protein gene product
Gαo	G-protein subunit α0
Gαi2	G-protein subunit αi2
Gγ8	G-protein subunit γ8
NSE	Neuron-specific enolase
CYK8	Cytokeratin 8
TUB	tubulin
PV	parvalbumin
OMP	Olfactory marker protein
LEA	*Lycopersicon esculentum* lectin
UEA	*Ulex europaeus* agglutinin
VVA	*Vicia villosa* agglutinin
SBA	*Glycine max lectin*
PHL	*Phaseolus vulgaris* lectin
ECL	*Erythrina cristagalli* lectin
LCA	*Lens culinaris* agglutinin
